# Advances in the Pathogenesis and Treatment of Resistant Hypertension

**DOI:** 10.3390/ijms241612911

**Published:** 2023-08-18

**Authors:** Jill Dybiec, Julia Krzemińska, Ewa Radzioch, Magdalena Szlagor, Magdalena Wronka, Ewelina Młynarska, Jacek Rysz, Beata Franczyk

**Affiliations:** 1 Department of Nephrocardiology, Medical University of Lodz, ul. Zeromskiego 113, 90-549 Łódź, Poland; jill.dybiec@gmail.com (J.D.); julia.krzeminska1@gmail.com (J.K.); ewa.m.radzioch@gmail.com (E.R.); szlagor.magdalena@gmail.com (M.S.); wronkam96@gmail.com (M.W.); bfranczyk-skora@wp.pl (B.F.); 2 Department of Nephrology, Hypertension and Family Medicine, Medical University of Lodz, ul. Żeromskiego 113, 90-549 Łódź, Poland; jacek.rysz@umed.lodz.pl

**Keywords:** hypertension, blood pressure measurement, secondary hypertension, renal denervation, antihypertensive drugs, resistant hypertension

## Abstract

Hypertension is a prevalent chronic disease associated with an increased risk of cardiovascular (CV) premature death, and its severe form manifests as resistant hypertension (RH). The accurate prevalence of resistant hypertension is difficult to determine due to the discrepancy in data from various populations, but according to recent publications, it ranges from 6% to 18% in hypertensive patients. However, a comprehensive understanding of the pathogenesis and treatment of RH is essential. This review emphasizes the importance of identifying the causes of treatment resistance in antihypertensive therapy and highlights the utilization of appropriate diagnostic methods. We discussed innovative therapies such as autonomic neuromodulation techniques like renal denervation (RDN) and carotid baroreceptor stimulation, along with invasive interventions such as arteriovenous anastomosis as potential approaches to support patients with inadequate medical treatment and enhance outcomes in RH.

## 1. Introduction

**Figure 1:**
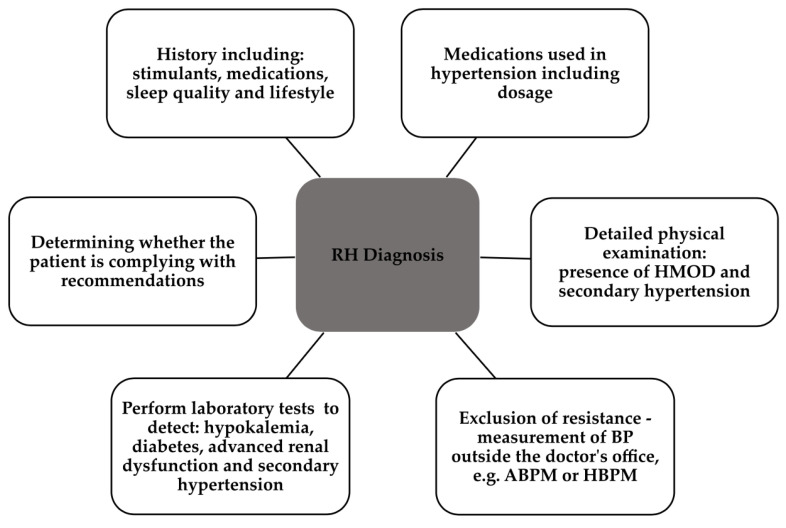
Detailed information collected from the patient to make a correct diagnosis [8]. RH, resistant hypertension; HMOD, hypertension-mediated organ damage; ABPM, ambulatory blood pressure monitoring; HBPM, home blood pressure monitoring.

Hypertension is one of the leading chronic diseases, ranking high among risk factors for cardiovascular (CV) death in patients. About one billion patients struggle with it, which poses quite a challenge to modern medicine [1,2,3]. A particularly severe form of hypertension is its variant, resistant hypertension (RH), which we define as high blood pressure (BP) that does not reach <140 mm Hg and/or <90 mm Hg despite the use of triple-drug therapy, containing drugs from different classes at maximum tolerated doses including a diuretic [1,4,5,6,7,8]. One of the criteria for identification is that the patient requires a supply of three BP-lowering drugs including a diuretic. In this definition, it is imperative that we exclude patients with white coat syndrome, masked hypertension, or lack of patient compliance [9]. Patients with RH are at higher risk for cardiovascular disease (CVD), chronic kidney disease, or diabetes. In addition, they have a significantly increased risk of organ complications and death from CV causes [2]. Many factors can be the cause of RH, but hypervolemia, medications such as nonsteroidal anti-inflammatory drugs (NSAIDs), glucocorticosteroids or antidepressants, undiagnosed secondary hypertension or excessive sympathetic nervous system activity due to chronic stress or pain should be considered as the most important [10]. The main limitation of the definition of RH that needs to be mentioned is the lack of distinction between pseudo-RH and true hypertension, which is why the correct diagnosis of these patients is so important. The inability to exclude the previously mentioned contributors to elevated BP is the reason for the terminology ATRH (apparent treatment-resistant hypertension) [11]. Thus, the Figure 1 illustrates the six most important elements necessary for the diagnosis of RH [8].

**Figure 2:**
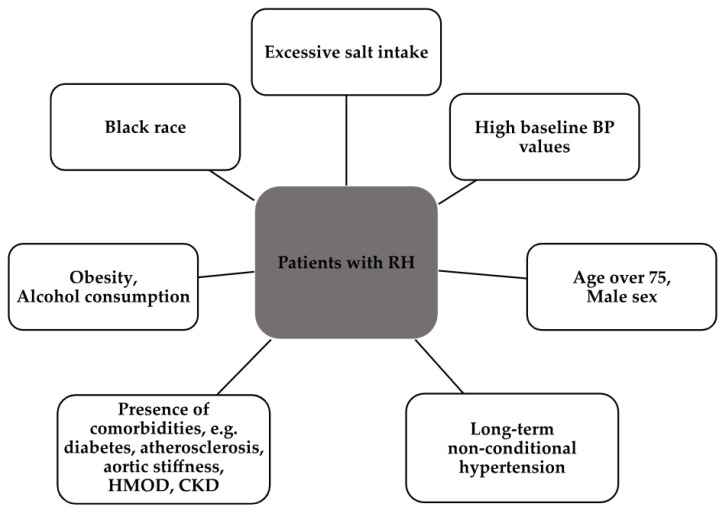
The most common characteristics observed in RH patients [8]. CKD, chronic kidney disease.

It is important that in patients with inadequate BP control, either ambulatory blood pressure monitoring (ABPM) or home blood pressure monitoring (HBPM) should be checked in patients with confirmed normal compliance. Figure 2 shows the most common characteristics observed in RH patients [8].

## 2. Epidemiology

According to many studies, the estimated range of RH prevalence is not conclusive due to varieties in populations and calculation methods [1,12,13,14,15,16]. An AHA (American Heart Association) statement declared ATRH prevalence to be between 12 and 18% [1], although German Health Examination Survey data suggest that ATRH refers to 6.8% of treated aware patients with hypertension [12]. De la Sierra et al. study reported that approximately 40% of patients with apparent RH had white coat RH [13]. European guidelines demonstrate the occurrence of true RH to be <10% of patients with hypertension [1]. The latest meta-analyses claim the presence of RH to be at the level of 10.6% [14] and 16.3% [15] in hypertensive patients. According to the Polish Society of Hypertension guidelines, in Poland, RH affects 10–13% of patients treated for hypertension [16]. The Pol-Fokus study suggests that RH may affect up to ~30% of hypertensive patients under the care of cardiology specialists [17].

Due to the analysis of data from the Scandinavian clinical program, initial higher BP, male gender, diabetes, left ventricular hypertrophy, increased body mass index (BMI), and higher alcohol consumption are the risk factors of RH development [18]. ATRH is also positively associated with diabetes [12]. Considering these factors, the most effective method of primary hypertension prevention is to avoid or delay the development of hypertension by lifestyle modification, especially obesity prevention and increasing physical activity.

The efficiency of antihypertensive treatment is variable, which is confirmed by the differences in the results of studies on arterial hypertension. This effectiveness is lower in patients with comorbidities such as diabetes or chronic kidney disease [10]. Most patients with RH carry a high CV risk [1,17].

Epidemiological studies on the occurrence of apparent and true RH are necessary to more precisely define the frequency of these types of hypertension and better assess this problem in the general population. The RH guidelines focus on the white coat effect, compliance, and pharmacological adherence as components that need to be considered when diagnosing RH [19] to exclude pseudo-RH. The high percentage of patients with RH among patients with arterial hypertension suggests considering an appropriate pharmacological and interventional strategy dedicated to this group of patients to reduce the very high CV risk.

## 3. Target Blood Pressure Values

The overarching goal of hypertension treatment is to lower BP values so as to reduce the risk of CV events [20,21]. The higher the risk, the greater the benefit for patients with lower target BP values [20]. There are two types of guidelines for classifying hypertension at different BP cutoff values. According to the American College of Cardiology/American Heart Association (ACC/AHA), hypertension is identified at values equal to or higher than systolic blood pressure (SBP) 130 and/or diastolic blood pressure (DBP) 80 mmHg; moreover, it distinguishes categories of normal BP, elevated BP, and two stages of hypertension. On the other hand, the European Society of Cardiology/European Society of Hypertension (ESC/ESH) considers values equal to or greater than 140 for SBP and/or 90 mmHg for DBP to be a diagnosis of hypertension; in addition, it distinguishes classes of optimal BP, normal BP, high normal BP, three classes of hypertension, and isolated systolic hypertension [22].

**Figure 3:**
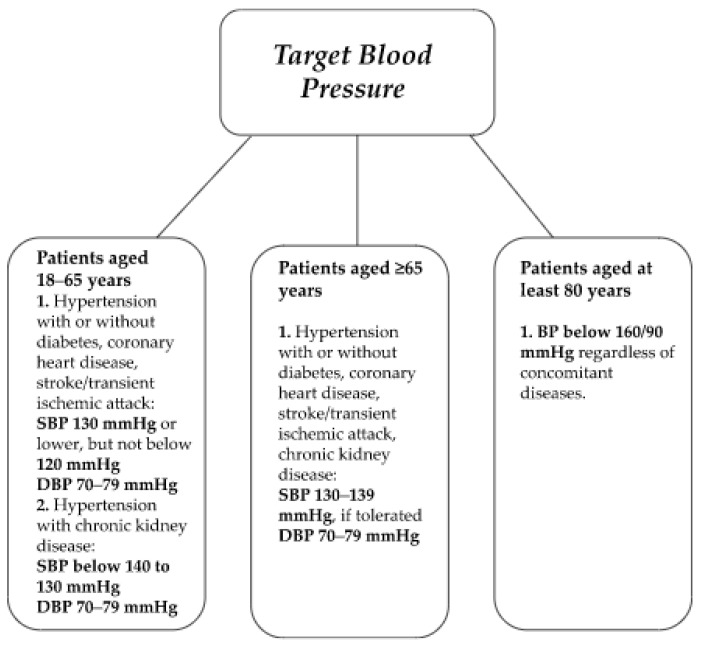
BP targets by age and concomitant diseases based on European Society of Cardiology/European Society of Hypertension guidelines [22,23]. SBP, systolic blood pressure; DBP, diastolic blood pressure.

Target BP values vary depending on age and concomitant diseases [23]. According to the ESC/ESH, BP values for adults should be below 140/90 mmHg, reaching a target of less than 130/80 mmHg for patients aged 18–65 [22,23]. Despite the set BP targets, it should be lowered individually for each patient, taking care to ensure safety, good tolerance and avoid adverse effects [22]. Figure 3 illustrates the target BP values depending on age and comorbidities [22,23].

## 4. Diagnostics of RH

### 4.1. Accurate Blood Pressure Measurement

**Figure 4:**
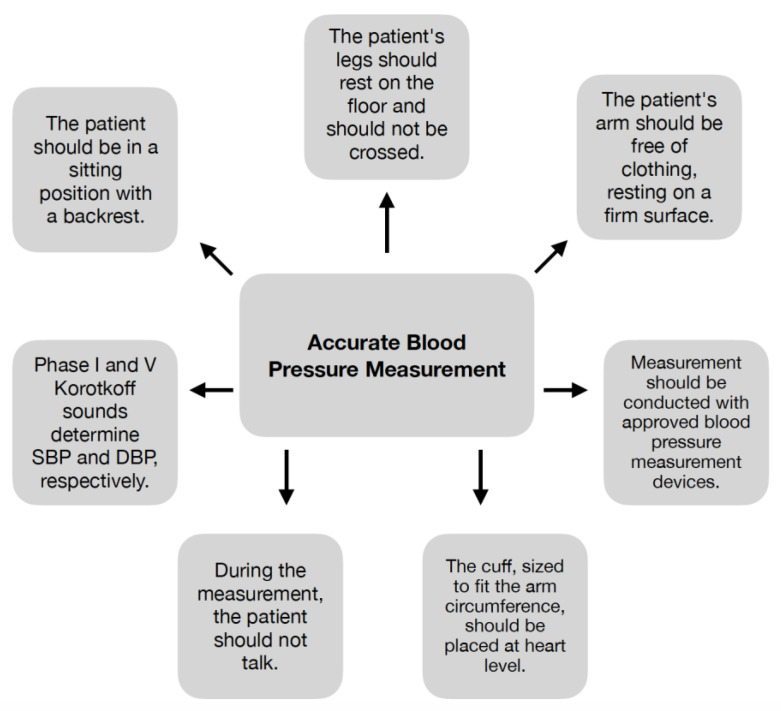
Principles of accurate BP measurement [8,24,26,27].

The basis for diagnosing RH is a properly performed BP measurement [24]. However, studies show that not following current guideline recommendations is a common mistake made during both office and HBPM [25]. Many factors can affect the outcome of a measurement, such as preparation for the examination, the technique used to perform the procedure, and the selection of the cuff [24]. Preparation for BP measurement includes cessation of coffee, tobacco, and alcohol consumption at least one hour before the measurement [26], as well as emptying the bladder [24,26]. The patient should also be asked about the consumption of medications or other substances that can affect BP values [26]. After a 5 min rest in a sitting position, in a quiet room with a comfortable temperature, the measurement can proceed [8,24,26]. The principles of accurate BP measurement are shown in Figure 4 [8,24,26,27].

A standard BP cuff is 12–13 cm wide and 35 cm long [8]. A properly selected cuff is one whose length is ≥80% and width is ≥40% of the arm circumference [24]. During the first appointment, measurements are taken on both arms [8]. When the results from the two upper limbs differ, subsequent measurements are taken on the limb where higher BP values were recorded [8,26]. BP should be checked three times, keeping a 1–2 min interval, and take the average of the last two results, discarding the first measurement [8,24].

One of the causes of a misdiagnosis of RH is incorrect BP measurement [8,24]. Common mistakes include a cuff worn over clothing, a cuff size that is too small, crossed legs, or a hanging arm [27].

Despite the availability of various arm cuff sizes, BP measurement in obese individuals with a large arm circumference can be problematic [28,29]. In addition to the upper arm cuff, measurement techniques are available at the forearm, wrist, or finger; however, their diagnostic accuracy appears uncertain. Irving et al. [28] concluded that, taking a properly fitted arm cuff as a reference, BP measurement using the method on the wrist held at heart level has the highest accuracy compared to the other methods. When taking measurements in obese individuals with a significant arm circumference, an alternative to an undersized arm cuff may be to measure with a wrist device [28]. Similar conclusions were reached by Mostafa et al. [29], who compared the accuracy of BP measurements using oscillometric arm, forearm, or wrist devices to invasive measurements in obese patients scheduled for bariatric surgery. A total of 40 patients were studied and showed the highest correlation of measurements between the invasive technique and the wrist-based oscillometric technique [29]. However, Zweiker et al. [30] showed a significant difference in BP values in wrist measurements using the oscillometric method and the auscultatory method, considered the gold standard. They maintain that measurements using the auscultatory method using an upper arm cuff should continue to remain the standard, as wrist measurements have low reliability [30].

For a correct diagnosis of RH, it is necessary to measure BP out of office to exclude the white coat effect [24]. According to a study by de la Sierra et al. [13], out of 8295 patients diagnosed with RH, as many as 37.5% had white coat resistance. The term “white coat syndrome” includes: (1) the white coat effect, (2) white coat hypertension, and (3) masked hypertension. The white coat effect refers to treated patients with hypertension who have an increase in SBP > 20 mmHg and DBP > 10 mmHg during office measurement in relation to ABPM or HBPM. The correlation between the occurrence of target organ damage and the white coat effect in patients with mild-to-moderate hypertension (including patients with RH) is weak. White coat hypertension is defined as a BP elevation ≥ 140/90 mmHg found at least three times in an untreated hypertensive patient with a mean 24 h ABPM <135/85 mmHg. It predisposes to the development of sustained hypertension, target organ damage, and increased CV risk. Masked hypertension is a condition in which normal BP values are observed in the office, whereas in ABPM or HBPM, they reach ≥130/80 mmHg and ≥135/85 mmHg, respectively [31]. It is estimated to affect about 15% of normotensive patients when measured in the office [8]. Masked hypertension increases the risk of CV [7,8], as well as the risk of renal incidents in patients with diabetes [8]. To determine the presence of any of the conditions belonging to white coat syndrome, BP measurements must be taken outside the doctor’s office; thus, HBPM or ABPM should be performed [8,31]. Moreover, they are a more accurate predictor of the occurrence of hypertension-mediated organ damage, or CV events, than office measurements [8].

Other reasons for a misdiagnosis of RH include poor adherence to medical recommendations (described in Section 4.3), significant calcification of the brachial artery, or a doctor’s therapeutic inertia [8].

### 4.2. Secondary Hypertension Screening

Due to the increased risk of CV complications in patients with RH, identifying the causes of antihypertensive treatment resistance is crucial. Secondary causes account for 5–10% of RH cases [10]. Secondary hypertension should be considered in electrolyte disturbances (for example hypokalemia), renal pathologies (such as parenchymal diseases or vascular hypertension), and hypertension in patients under 40 years old. Furthermore, the diagnostics may be extended in the cases of hypertension with sudden onset or organ damage disproportionate to the presence of hypertension. The symptoms that may indicate aortic coarctation, Cushing’s syndrome, or pheochromocytoma should be studied when diagnosing secondary hypertension as well [8,19,32]. In fact, obstructive sleep apnea (OSA) is also a common RH cause with a prevalence rate between 70% and 90% [33,34]; patients should be screened for the symptoms of that disorder. The most common are loud snoring, periods of apnea during sleep, and excessive daytime sleepiness [35]. It has been shown that an increased secretion of aldosterone may contribute in OSA patients to the development of resistance to antihypertensive therapy [36]. Psoriasis-related systemic inflammation can also contribute to resistance [16].

When investigating secondary causes of RHT, it is critical to exclude the pseudo-RH causes such as brachial artery calcification, which leads to the closure of the forearm arteries when the BP cuff inflates. That condition is very common, especially in older patients. Other causes of pseudo-RH that need to be ruled out are poor adherence to prescribed medicines and the white coat effect [8]. In the case of white coat syndrome, ABPM is recommended. ABPM detected white coat effect hypertension in 44% of 286 patients with suspected RH [10]. Untreatable high BP may also be related to drugs including oral contraceptives, NSAIDs, immunosuppressive agents, herbal supplements, stimulants [19], and also rare genetic etiologies, especially in young patients [1]. These factors should also be considered during the diagnosis of RH.

The latest guidelines recommend RH evaluation with routine screening for secondary hypertension, including medical history, physical exam, and 24 h BP recording (ABPM) [1]. Further tests involve blood and urine tests, abdominal ultrasound, and echocardiography for aortic coarctation. Specialist center diagnostics confirm suspicions with abnormal results. The choice of instrumental examination, such as ultrasound, computer tomography (CT), and magnetic resonance imaging (MRI), depends on diagnostic challenges and patient characteristics. Ultrasonography with duplex Doppler is commonly used for renal vascular diseases [8]. Other imaging modalities serve as alternatives. Renal angiography is favorable if other methods fail to diagnose secondary hypertension.

To estimate the OSA risk, various questionnaires are used. The commonly used ones include the Berlin questionnaire, Epworth Sleepiness Scale, and the STOP-Bang questionnaire [35]. To confirm OSA, clinicians should proceed overnight with oximetry and polysomnography [1] or, according to European guidelines, ambulatory polygraphy [8]. In cases of suspected endocrine diseases, other additional specific-to-individual-conditions tests, such as thyroid function tests, 24 h urinary-free cortisol, or 24 h urinary fractionated metanephrines, should be performed.

The most common endocrine cause of RH is hyperaldosteronism, with the prevalence of primary aldosteronism from 5 to 15% in the hypertension population [8]. Primary aldosteronism should be suspected in patients with RH characterized by significantly elevated BP values in ABPM (especially during the night), impaired glucose tolerance, diabetes, left ventricular hypertrophy, or microalbuminuria [37]. Spontaneous hypokalemia and muscle weakness may also indicate primary hyperaldosteronism [16]. When screening for hyperaldosteronism, it is recommended to perform plasma aldosterone and renin sampling and aldosterone/renin ratio. It is a vital aspect that hypokalemia can depress aldosterone levels so it needs to be corrected before measurements [8].

The early detection of secondary hypertension enables prompt intervention to mitigate its effects and prevent permanent changes in the body. That is particularly crucial in younger patients, as interventions later in life are less likely to be cured due to the permanent vascular and organ damage caused by chronic hypertension in these individuals.

### 4.3. Drug Adherence Estimation

Inadequate drug adherence is a significant problem in the treatment of AH. It is a common cause of poor BP control, as well as pseudo-RH (≤50% of patients). It usually occurs as irregular medication intake and early treatment discontinuation. The consequence of nonadherence is an increased risk of CV events [8]. There are several methods for drug adherence evaluation. These include measuring drug concentrations in the patient’s serum or urine and measuring BP after supervised drug intake [8,26]. Methods such as questionnaires are not recommended [8].

A post hoc analysis by Kario et al. [38] aimed to explain the reasons for the discrepancies in the results of the REQUIRE study, compared to other studies determining the effectiveness of ultrasound renal denervation (uRDN). The REQUIRE study, unlike other studies (RADIANCE-HTN SOLO, RADIANCE-HTN TRIO, and RADIANCE II), did not show a reduction in 24 h ambulatory SBP in RH patients undergoing uRDN, compared to the sham procedure. A post hoc analysis was conducted to assess whether these results were influenced by medication adherence. It was proven that almost half of RH patients showed poor adherence to medical recommendations. In contrast, the reason for the BP reduction in the sham group was improved drug adherence during the study. Moreover, it was shown that the uRDN procedure was most effective in patients with good drug adherence both at the beginning and throughout the study period. This shows that medication adherence is also crucial for RH patients in the context of device-based therapies [38].

Similar results regarding the percentage of nonadherence to medication were provided by a meta-analysis by Bourque et al. [39], where nonadherence was 37%. Interestingly, direct methods such as urine tests or directly observed therapy, with which nonadherence was diagnosed at 46%, were much more effective in assessing drug adherence. On the contrary, indirect methods such as questionnaires or pill counts proved to be unreliable, and with their use, the nonadherence rate reached only 20% [39].

Coinciding with the aforementioned are the results of a meta-analysis by Lee et al. [40], where antihypertensive medication (AHM) nonadherence was estimated at 27–40% and did not improve between 2010 and 2020. Moreover, higher adherence was proven in Western countries and areas with high population density. However, the researchers emphasized that the high heterogeneity may have limited the reliability of the meta-analysis [40].

Adherence to AHM recommendations is also important in terms of the occurrence of CVD events. Feng et al. [41] proved that an increase in medication adherence is associated with a decreased risk of CVD incidents, which demonstrates the long-term benefits of good drug adherence.

The reasons for such high nonadherence include the high cost of treatment, the complicated dosing of AHM, the occurrence of numerous side effects, or therapeutic inertia, among others [24].

Articles summarizing studies evaluating treatment adherence are presented in Table 1 [38,39,40,41].

There are several ways to improve drug adherence. These include simplifying therapy, i.e., preferring long-acting drugs, avoiding the complex dosing regimen of AHM, and using single-pill combinations as often as possible. In addition, it is important to avoid the side effects of the drug and to make therapy as compatible as possible with the patient’s financial capabilities. It is also essential to try to look for ways to improve adherence that are individually tailored to the patient, to motivate and encourage them to take care of their health [8].

Recent studies suggest that telemetry may be important in improving drug adherence [8]. High hopes are now being placed on modern solutions such as clinical decision support systems to help provide clear and patient-tailored treatment recommendations [42]. Smartphone apps, whose mechanism of action is based on the patient’s behavioral self-monitoring, can also be helpful. Smartphone apps, when co-administered with personalized medical advice, have been shown to have a clinically significant effect on lowering BP [43].

## 5. Pharmacological Treatment

It is very important to start the whole treatment process first with nonpharmacological methods that will, of course, only gently support pharmacological therapy. It is well known that a healthy lifestyle is a very important factor affecting quality of life and health, and in this case, BP values [44]. Patients should therefore, first of all, limit their dietary salt supply to 4–6 g/day and practice physical activity. [8,44]. The best form of sport that lowers both SBP and DBP is regular aerobic exercise, including walking or running on a treadmill. Moreover, in addition to aerobic training, resistance training has been proven to be a simultaneously recommended workout that affects BP decrease [24,44]. The researchers came to an interesting conclusion in a pilot study that tested the effect of 1 h of exercise in a swimming pool, where the water was 32 degrees, and training was carried out three times a week for a period of 14 days. The study showed a decrease of 9 mmHg in 24 h ambulatory measurements. The authors concluded that the hypotensive effect was achieved, in addition to reducing sympathetic activity, through vasodilation or increasing the release of nitric oxide. Another element affecting the reduction in pressure values is, of course, the loss of adipose tissue, especially that located viscerally, in the kidney area [6].

Pharmacological treatment consists of a group of ≥3 hypotensive drugs. The classic regimen consists of the use of:

1. A blocker of the RAA system, with special emphasis on angiotensin-converting enzyme inhibitors (ACEis) and angiotensin receptor blockers (ARBs);
2. A long-acting calcium channel blocker (CCB), most commonly amlodipine;
3. A long-acting thiazide diuretic, i.e., indapamide or chlorthalidone.

We also have other drugs like aldosterone synthase inhibitors or dual endothelin antagonists to choose from [45].

**Figure 5:**
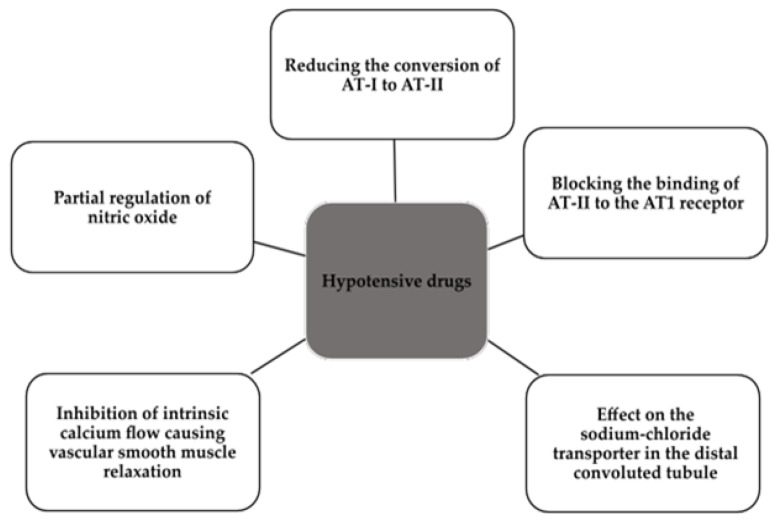
Mechanism of action of drugs used in patients with RH [46,47,48]. AT-I, angiotensin-I; AT-II, angiotensin-II; AT1, angiotensin receptor 1.

These three large groups of drugs are complementary in their mechanisms affecting the lowering of BP values by which, through the choice of doses and frequency of administration, they should give due therapeutic effect and reduce the risk of death [4,8]. The mechanisms of the three groups of BP-lowering drugs in patients with RH are included in Figure 5 [46,47,48]. Recent studies show the possibility of using chlorthalidone in patients with chronic kidney disease, so we do not have to reduce the dose due to low glomerular filtration rate (GFR), and this therapy clearly improves blood pressure values compared to placebo [49].

The PATHWAY-2 (Prevention and Treatment of Hypertension With Algorithm-Based Therapy) trial showed a significant benefit when patients were supplemented with another drug, spironolactone, a mineralocorticoid receptor antagonist, in addition to the above-mentioned drugs. It is worth adding that due to side effects such as gynecomastia, impotence, and menstrual disorders, not all patients will tolerate mineralocorticoid receptor antagonists (MRAs) well. Due to the small number of studies on patients with renal impairment, we can use this drug for those with eGFR ≥45 mL/min and serum potassium levels ≤4.5 mmol/L [4,8]. An alternative to spironolactone is amiloride, bisoprolol, or doxazosin, especially in patients with contraindications or intolerance to spironolactone. A group of new BP-lowering drugs is still under investigation [8].

A group worth paying special attention to because of the increased risk of dehydration and orthostatic hypotension are the elderly. As they age, their physiological mechanisms weaken, and both thirst and the accumulation of primary urine in the kidneys decrease. Their total body water decreases, which consequently leads to lower fluid stores. In addition to physiological causes, we can add risk factors such as cognitive impairment, dysphagia, and deliberately reduced fluid intake, for example, due to fear of incontinence. Furthermore, frequent use of diuretics leads to an even further intensification of the emptying of fluid stores, further aggravating dehydration [50]. Orthostatic hypotension is very common among the elderly. It depends on the action of the autonomic nervous system responding to baroreceptors, whose sensitivity is impaired in the elderly, most likely due to atherosclerosis. Moreover, reduced physical activity, lack of exercise, delayed heart rate recovery on standing, frailty, arterial stiffness and cognitive impairment are other risk factors. However, it is the drug that is the most common reason for the appearance of cases of orthostatic hypotension in emergency departments. Among these drugs, we can include, for example, selective serotonin reuptake inhibitors and selective norepinephrine reuptake inhibitors, but also, importantly, diuretics [51].

## 6. Nonpharmacological Treatment

### 6.1. Renal Denervation

Renal denervation (RDN) is a minimally invasive, investigational procedure that has emerged as a potential therapeutic option for patients with RH. Its mechanism of action includes interrupting the renal sympathetic activity by destroying sympathetic nerves localized close to the renal arteries. Activation of the efferent renal sympathetic nerves leads to renin secretion, sodium absorption as well as increased renal vascular resistance. Consequently, it contributes to the pathogenesis of hypertension. The result of RDN is a reduction in sympathetic activity and, therefore, a decrease in renin activity and an increase in renal blood flow [52]. RH is a primary indication for RDN. In clinical practice, it is important to confirm RH (by excluding white coat hypertension in 24 h AMPM) and to expel secondary causes of hypertension before the procedure.

Early trials on RDN are focused on severe hypertension, with SBP over 160 mmHg [52,53,54]. In the SYMPLICITY HTN-2 trial, Murray et al. [53] revealed that RDN is safe and provides a lasting reduction in BP to 1 year. However, the SYMPLICITY HTN-3 trial [54] proved to be a failure. This might be due to the greater severity of hypertension, which increases the number of confounding factors.

More recent studies have focused on patients with lower BP values [55,56,57]. The results of SPYRAL HTN-OFF MED [55] confirmed the efficacy of RDN in lowering BP in the absence of medications. A similar study was performed by Azizi et al. [56]. RDN proved to be beneficial in lowering ambulatory BP in this group of patients. Furthermore, the SPYRAL HTN-ON MED trial [57] showed remarkably lower values of BP at 6 months in the RDN group. A summary of the above-mentioned studies is presented in Table 2.

### 6.2. Carotid Baroreceptor Stimulation

The aim of baroreflex activation therapy (BAT) devices is to artificially strain the carotid baroreceptor and, as a consequence, induce a decrease in BP. Carotid baroreceptor stimulation (CBS) was initially associated with a high number of adverse incomes, mainly related to periprocedural complications due to the implantation of electrodes around both carotid arteries. However, the procedure has developed and shown promising effects in recent years.

The first-generation CBS system called Rheos consists of two electrodes implanted bilaterally in the region of the carotid sinus and conducted between the heads of the sternocleidomastoid muscle to a pulse generator. Rheos has been improved to a second-generation device, which is Barostim neo™ [58]. It differs from Rheos in the size of the pulse generator and the number of required electrodes. The significant advantages of this device are less extensive neck dissection without external carotid artery exposure as well as one-sided implantation with the single-button electrode sutured to the carotid sinus.

The first study assessing BAT with a long-term implantable device was the DEBuT-HT trial (Device-Based Therapy in Hypertension Trial) [59]. CVRx Rheos^®^ device was used. After 3 months of baroreceptor stimulation, a reduction in SBP of 21 mm Hg and DBP of 12 mmHg was achieved. The reduction in BP was even greater after 12 and 24 months of treatment. The Rheos Pivotal Trial [60], a double-blind randomized trial, resulted in a sustained reduction in BP. At 12-month follow-up, in both groups, the average reduction was 25 mm Hg. Similar outcomes were revealed in the Barostim neo trial [58]. At 3 months, BP reduction was, on average, 26 mm Hg and remained stable for the next 3 months.

Clinical trials on BAT are still in progress. The MobiusHD™ is a device undergoing investigation that is not based on electrical stimulation [61]. This stent-like device activates the baroreceptor by stretching the carotid sinus. It is promising to be a more long-term solution than the previous generations.

### 6.3. Arteriovenous Fistula

Arteriovenous anastomosis is another of the invasive treatments for hypertension [62]. It has been shown that the use of an arteriovenous fistula (AVF) in patients with RH is able to significantly reduce BP compared to pharmacotherapy alone [63]. The hypotensive effect is achieved by adding a high-resistance venous segment to the central arteries, and the mechanism of action of such a fistula is the flow of blood from the arterial to the venous vessels, resulting in a decrease in peripheral resistance, relieving pressure on the left ventricle and increasing the right ventricular workload [62,63]. The advantage of this method of device treatment is its reversibility because, unlike denervation or ablation, the connector can be closed, blocking its effect [64]. Unfortunately, this method has its side effects such as symptomatic venous constriction, lower extremity edema, right ventricular dysfunction, and increased pulmonary pressure [62,63].

A meta-analysis by Scholz et al. [63] evaluated the effects of AVF generation and ligation on BP parameters in patients with end-stage renal disease requiring hemodialysis. AVF generation led to a significant decrease in SBP (8.7 ± 28.5 mm Hg), DBP, and MAP, while AVF closure led to an increase in BP [63]. Mathew et al. [65] conducted a similar study and showed that patients with end-stage renal disease before dialysis with AVF had a small reduction in BP over 12 months of follow-up, with a greater reduction noted for DBP [65]. In contrast, Faul et al. [66] evaluated the efficacy of a percutaneously created iliac AVF in patients with COPD and hypertension. Twenty-four patients already treated for hypertension with mainly two drugs underwent fistula surgery and were observed for one year. After this period, there was a decrease in both SBP (from 145 mmHg to 132 mmHg) and DBP (from 86 mmHg to 67 mmHg), and there was no correlation between the severity of changes in BP values and other factors such as gender, age, or severity of COPD. Moreover, there was a 34% reduction in patients with SBP values higher than 140 mmHg, so these results suggest that AVF may be an effective tool in the treatment of RH [66]. Lobo et al. [64] also reported significant reductions in SBP and DBP in both office and ABP measurements among severely hypertensive patients with arteriovenous anastomosis after a 12-month analysis. Importantly, BP reduction occurred in both daytime and nighttime measurements. However, some patients experienced adverse effects, including lower extremity edema in 29%, which, however, could be successfully treated by stenting or venoplasty [64]. A study based on the same group of patients by Lobo et al. [67] evaluated the safety and efficacy of an arteriovenous linker in patients with RH. After a 6-month follow-up, there was a significant reduction in mean SBP and DBP in the intervention group. Interestingly, the same significant reduction in BP was noted both in patients after RDN and in those without it. In conclusion, this type of therapy appears to be effective and safe, but further research is needed to determine whether this method can be successfully used in patients who cannot or do not wish to receive pharmacological treatment [67]. Randomized trials that measure BP with methods more precise than office measurements such as 24 h BP monitoring would also be useful. In addition, studies focusing on the long-term effects of AVF generation would be beneficial [63]. Table 3 summarizes the studies discussed [63,64,65,66].

## 7. Conclusions

To properly diagnose RH, BP measurement should be performed meticulously following the recommendations of current guidelines. It is also necessary to exclude factors that may contribute to the misdiagnosis of RH, so-called pseudo-RH.

In the diagnosis of RHT, it is crucial to consider the presence of secondary causes that contribute to its development. Early detection of secondary HT, particularly in younger patients, holds the potential to prevent irreversible structural and functional changes in organs, thus mitigating the risk of organ failure.

The pharmacological treatment of RH is complex. The main groups of drugs are blockers of the RAA system, long-acting calcium channel blockers, and long-acting thiazide diuretics, which are combined in polytherapy. In addition to pharmacological treatments, all patients at every stage of therapy should take care of the principles of a healthy lifestyle, i.e., limit salt intake, increase physical activity, and reduce body fat. Furthermore, the use of autonomic neuromodulation therapies in RH treatment has become very promising recently. Clinical trials on device-based therapies are still ongoing.

## Figures and Tables

**Table 1:** Drug adherence estimation [38,39,40,41].

Authors	Kario et al. [38]	Bourque et al. [39]	Lee et al. [40]	Feng et al. [41]
Year	2023	2023	2022	2021
All patients	58	71,353	27,785,595	2,769,700
Study design	Post hoc analysis	Meta-analysis	Meta-analysis	Meta-analysis
Patient characteristics	RH patients aged 20–75 years old.	RH patients aged ≥18 years old; uncontrolled with ≥3 drugs or controlled with ≥4 drugs.	AH patients with a mean age of 57; 42.9% men.	AH patients ≥18 years old at baseline, without a history of CVD events.
Aim of the study	Drug adherence at baseline and after 3 months postprocedure (uRDN).	Determine the overall prevalence of nonadherence and evaluate the effect of the method of assessment on this estimate.	Explore global epidemiology, regional differences, and trends in AHM nonadherence.	Determine the association between AHM adherence and risk of CVD events in patients with AH.
Methods	Urine sample testing for AHM or their metabolites to assess drug adherence.	MEDLINE, EMBASE, Cochrane, CINAHL, and Web of Science (databases from inception to November 2020) were searched.	Multiple medical databases and clinicaltrials.gov were searched for articles (data from 2010 to 2020).	Two databases (PubMed and Embase) from 1974 to 15 December 2019 were searched.
Drug adherence evaluation	45% of RH patients had poor medication adherence.	The overall incidence of nonadherence was 37% (20% for indirect methods and 46% for direct methods).	AHM nonadherence did not improve between 2010 and 2020 and remained at 27–40%.	Each 20% increase in AHM adherence was associated with a 13% reduced risk of CVD events.

RH, resistant hypertension; AH, arterial hypertension; CVD, cardiovascular disease; uRDN, ultrasound renal denervation; AHM, antihypertensive medication.

**Table 2:** Summary of studies on RDN [53,54,55,56,57].

Trail	Year	Study Patients’ Office SBP	Study Patients’ Office DBP	Study Patients’ Ambulatory BP	Results	Reference
SYMPLICITY HTN-2	2012	≥160 mm Hg (≥150 mm Hg if they had T2DM)	-	-	lasting BP reduction to 1 year	[53]
SYMPLICITY HTN-3	2014	≥160 mm Hg	-	-	no significant BP reduction	[54]
SPYRAL HTN-OFF MED	2017	150–180 mm Hg	≥90 mm Hg	SBP 140–170 mm Hg	significant BP reduction	[55]
RADIANCE-HTN SOLO	2018	-	-	SBP 135–170 mm Hg DBP 85–105 mm Hg	significant BP reduction	[56]
SPYRAL HTN-ON MED	2018	150–180 mm Hg	≥90 mm Hg	SBP 140–170 mm Hg	lasting BP reduction to 6 months	[57]

SBP, systolic blood pressure; DBP, diastolic blood pressure; BP, blood pressure; T2DM, type 2 diabetes mellitus.

**Table 3:** Summary of studies on AVF [63,64,65,66].

Authors	Scholz et al. [63]	Lobo et al. [64]	Mathew et al. [65]	Faul et al. [66]
Study design	Meta-analysis	Randomized controlled trial	Comparative study	Clinical trial
All patients	412	83	5095	24
Patient category	Patients with ESRD, on dialysis or before dialysis, as well as post-transplant patients who were scheduled to receive an AVF or were already on an AVF implantation procedure.	Patients aged 18–80 years who maintained high BP values despite pharmacotherapy with three or more drugs.	Patients with ESRD before starting hemodialysis.	Patients with COPD and high BP values despite pharmacological treatment.
Effect on BP	AVF creation led to a significant reduction in SBP, DBP, and MAP, while AVF closure led to an increase in BP.	AFV caused a reduction in SBP and DBP.	A small reduction in BP over 12 months of follow-up in the intervention group was reported, with a greater reduction in DBP.	AFV caused a decrease in SBP and DBP.

ESRD, end-stage renal disease; AVF, arteriovenous fistula.

## Data Availability

The data used in this article were sourced from materials mentioned in the References section.

## References

[1] Lamirault G., Artifoni M., Daniel M., Barber-Chamoux N., Nantes University Hospital Working Group On Hypertension (2020). Resistant Hypertension: Novel Insights. Curr. Hypertens. Rev..

[2] Sarwar M.S., Islam M.S., Al Baker S.M., Hasnat A. (2013). Resistant hypertension: Underlying causes and treatment. Drug Res..

[3] Smith S.M. (2013). Epidemiology, prognosis, and treatment of resistant hypertension. Pharmacotherapy.

[4] Acelajado M.C., Hughes Z.H., Oparil S., Calhoun D.A. (2019). Treatment of Resistant and Refractory Hypertension. Circ. Res..

[5] Judd E., Calhoun D.A. (2014). Apparent and true resistant hypertension: Definition, prevalence and outcomes. J. Hum. Hypertens..

[6] Ozemek C., Tiwari S., Sabbahi A., Carbone S., Lavie C.J. (2020). Impact of therapeutic lifestyle changes in resistant hypertension. Prog. Cardiovasc. Dis..

[7] Egan B.M. (2015). Treatment Resistant Hypertension. Ethn. Dis..

[8] Williams B., Mancia G., Spiering W., Agabiti Rosei E., Azizi M., Burnier M., Clement D.L., Coca A., de Simone G., Dominiczak A. (2018). 2018 ESC/ESH Guidelines for the management of arterial hypertension. Eur. Heart J..

[9] Moreno H. (2022). Pseudo and resistant hypertension: A chaotic perspective. J. Clin. Hypertens..

[10] Doroszko A., Janus A., Szahidewicz-Krupska E., Mazur G., Derkacz A. (2016). Resistant Hypertension. Adv. Clin. Exp. Med..

[11] Nagarajan N., Jalal D. (2019). Resistant Hypertension: Diagnosis and Management. Adv. Chronic. Kidney Dis..

[12] Sarganas G., Neuhauser H.K. (2016). Untreated, Uncontrolled, and Apparent Resistant Hypertension: Results of the German Health Examination Survey 2008–2011. J. Clin. Hypertens..

[13] de la Sierra A., Segura J., Banegas J.R., Gorostidi M., de la Cruz J.J., Armario P., Oliveras A., Ruilope L.M. (2011). Clinical features of 8295 patients with resistant hypertension classified on the basis of ambulatory blood pressure monitoring. Hypertension.

[14] Achelrod D., Wenzel U., Frey S. (2015). Systematic review and meta-analysis of the prevalence of resistant hypertension in treated hypertensive populations. Am. J. Hypertens..

[15] Noubiap J.J., Nansseu J.R., Nyaga U.F., Sime P.S., Francis I., Bigna J.J. (2019). Global prevalence of resistant hypertension: A meta-analysis of data from 3.2 million patients. Heart.

[16] Tykarski A., Widecka K., Narkiewicz K. (2019). Zasady postępowania w nadciśnieniu tętniczym—2019 rok Wytyczne Polskiego Towarzystwa Nadciśnienia Tętniczego. Próba komentarza na temat zmian i ich zasadności. Nadciśnienie Tętnicze w Praktyce.

[17] Prejbisz A., Klocek M., Gąsowski J., Topór-Mądry R., Leśniak W., Kabat M., Czarnecka D., Kawecka-Jaszcz K., Narkiewicz K., Januszewicz A. (2015). Factors associated with resistant hypertension in a large cohort of hypertensive patients: The Pol-Fokus study. Pol. Arch. Med. Wewn..

[18] Gupta A.K., Nasothimiou E.G., Chang C.L., Sever P.S., Dahlöf B., Poulter N.R., ASCOT Investigators (2011). Baseline predictors of resistant hypertension in the Anglo-Scandinavian Cardiac Outcome Trial (ASCOT): A risk score to identify those at high-risk. J. Hypertens..

[19] Kim H.M., Shin J. (2023). Role of home blood pressure monitoring in resistant hypertension. Clin. Hypertens..

[20] Sudano I., Osto E., Ruschitzka F., von Eckardstein A., Binder C.J. (2022). Blood Pressure-Lowering Therapy. Prevention and Treatment of Atherosclerosis: Improving State-of-the-Art Management and Search for Novel Targets.

[21] Singh J.N., Nguyen T., Kerndt C.C., Dhamoon A.S. (2023). Physiology, Blood Pressure Age Related Changes. StatPearls.

[22] National Guideline Centre (UK) (2022). Evidence Review for Blood Pressure Targets: Hypertension in Adults (Update): Evidence Review J.

[23] Iqbal A.M., Jamal S.F. (2023). Essential Hypertension. StatPearls.

[24] Shalaeva E.V., Messerli F.H. (2023). What is resistant arterial hypertension?. Blood Press..

[25] Beger C., Mayerböck A., Klein K., Karg T., Schmidt-Ott K.M., Randerath O., Limbourg F.P. (2023). Current practice of blood pressure measurement in Germany: A nationwide questionnaire-based survey in medical practices. Blood Press..

[26] Jordan J., Kurschat C., Reuter H. (2018). Arterial Hypertension. Dtsch. Arztebl. Int..

[27] Flack J.M., Adekola B. (2020). Blood pressure and the new ACC/AHA hypertension guidelines. Trends Cardiovasc. Med..

[28] Irving G., Holden J., Stevens R., McManus R.J. (2016). Which cuff should I use? Indirect blood pressure measurement for the diagnosis of hypertension in patients with obesity: A diagnostic accuracy review. BMJ Open.

[29] Mostafa M.M.A., Hasanin A.M., Alhamade F., Abdelhamid B., Safina A.G., Kasem S.M., Hosny O., Mahmoud M., Fouad E., Rady A. (2020). Accuracy and trending of non-invasive oscillometric blood pressure monitoring at the wrist in obese patients. Anaesth. Crit. Care Pain. Med..

[30] Zweiker R., Schumacher M., Fruhwald F.M., Watzinger N., Klein W. (2000). Comparison of wrist blood pressure measurement with conventional sphygmomanometry at a cardiology outpatient clinic. J. Hypertens..

[31] Pioli M.R., Ritter A.M., de Faria A.P., Modolo R. (2018). White coat syndrome and its variations: Differences and clinical impact. Integr. Blood Press. Control..

[32] Whelton P.K., Carey R.M., Aronow W.S., Casey D.E., Collins K.J., Dennison Himmelfarb C., DePalma S.M., Gidding S., Jamerson K.A., Jones D.W. (2018). 2017 ACC/AHA/AAPA/ABC/ACPM/AGS/APhA/ASH/ASPC/NMA/PCNA Guideline for the Prevention, Detection, Evaluation, and Management of High Blood Pressure in Adults: A Report of the American College of Cardiology/American Heart Association Task Force on Clinical Practice Guidelines. Hypertension.

[33] Muxfeldt E.S., Margallo V.S., Guimarães G.M., Salles G.F. (2014). Prevalence and associated factors of obstructive sleep apnea in patients with resistant hypertension. Am. J. Hypertens..

[34] Lloberes P., Lozano L., Sampol G., Romero O., Jurado M.J., Ríos J., Untoria M.D., Tovar J.L. (2010). Obstructive sleep apnoea and 24-h blood pressure in patients with resistant hypertension. J. Sleep Res..

[35] Sarathy H., Salman L.A., Lee C., Cohen J.B. (2022). Evaluation and Management of Secondary Hypertension. Med. Clin. N. Am..

[36] Bioletto F., Bollati M., Lopez C., Arata S., Procopio M., Ponzetto F., Ghigo E., Maccario M., Parasiliti-Caprino M. (2022). Primary Aldosteronism and Resistant Hypertension: A Pathophysiological Insight. Int. J. Mol. Sci..

[37] Florczak E., Prejbisz A., Szwench-Pietrasz E., Sliwiński P., Bieleń P., Klisiewicz A., Michałowska I., Warchoł E., Januszewicz M., Kała M. (2013). Clinical characteristics of patients with resistant hypertension: The RESIST-POL study. J. Hum. Hypertens..

[38] Kario K., Kai H., Nanto S., Yokoi H. (2023). Anti-hypertensive medication adherence in the REQUIRE trial: Post-hoc exploratory evaluation. Hypertens. Res..

[39] Bourque G., Ilin J.V., Ruzicka M., Hundemer G.L., Shorr R., Hiremath S. (2023). Non-Adherence is Common in Patients with Apparent Resistant Hypertension: A Systematic Review and Meta-Analysis. Am. J. Hypertens..

[40] Lee E.K.P., Poon P., Yip B.H.K., Bo Y., Zhu M.T., Yu C.P., Ngai A.C.H., Wong M.C.S., Wong S.Y.S. (2022). Global Burden, Regional Differences, Trends, and Health Consequences of Medication Nonadherence for Hypertension During 2010 to 2020: A Meta-Analysis Involving 27 Million Patients. J. Am. Heart Assoc..

[41] Feng Y., Zhao Y., Yang X., Li Y., Han M., Qie R., Huang S., Wu X., Zhang Y., Wu Y. (2022). Adherence to antihypertensive medication and cardiovascular disease events in hypertensive patients: A dose-response meta-analysis of 2,769,700 participants in cohort study. QJM.

[42] Mai A., Voigt K., Schübel J., Gräßer F. (2023). A drug recommender system for the treatment of hypertension. BMC Med. Inform. Decis Mak..

[43] Kassavou A., Wang M., Mirzaei V., Shpendi S., Hasan R. (2022). The Association Between Smartphone App-Based Self-monitoring of Hypertension-Related Behaviors and Reductions in High Blood Pressure: Systematic Review and Meta-analysis. JMIR Mhealth Uhealth..

[44] Ruilope L.M., Rodríguez-Sánchez E., Navarro-García J.A., Segura J., Órtiz A., Lucia A., Ruiz-Hurtado G. (2020). Resistant hypertension: New insights and therapeutic perspectives. Eur. Heart J. Cardiovasc. Pharmacother..

[45] Weldon S.M., Brown N.F. (2019). Inhibitors of Aldosterone Synthase. Vitam. Horm..

[46] Goyal A., Cusick A.S., Thielemier B. (2023). ACE Inhibitors. StatPearls.

[47] Burnier M., Bakris G., Williams B. (2019). Redefining diuretics use in hypertension: Why select a thiazide-like diuretic?. J. Hypertens..

[48] Elliott W.J., Ram C.V. (2011). Calcium channel blockers. J. Clin. Hypertens..

[49] Agarwal R., Sinha A.D., Cramer A.E., Balmes-Fenwick M., Dickinson J.H., Ouyang F., Tu W. (2021). Chlorthalidone for Hypertension in Advanced Chronic Kidney Disease. N. Engl. J. Med..

[50] Beck A.M., Seemer J., Knudsen A.W., Munk T. (2021). Narrative Review of Low-Intake Dehydration in Older Adults. Nutrients.

[51] Dani M., Dirksen A., Taraborrelli P., Panagopolous D., Torocastro M., Sutton R., Lim P.B. (2021). Orthostatic hypotension in older people: Considerations, diagnosis and management. Clin. Med..

[52] Schlaich M.P., Sobotka P.A., Krum H., Lambert E., Esler M.D. (2009). Renal sympathetic-nerve ablation for uncontrolled hypertension. N. Engl. J. Med..

[53] Esler M.D., Krum H., Schlaich M., Schmieder R.E., Böhm M., Sobotka P.A., Symplicity HTN-2 Investigators (2012). Renal sympathetic denervation for treatment of drug-resistant hypertension: One-year results from the Symplicity HTN-2 randomized, controlled trial. Circulation.

[54] Bhatt D.L., Kandzari D.E., O’Neill W.W., D’Agostino R., Flack J.M., Katzen B.T., Leon M.B., Liu M., Mauri L., Negoita M. (2014). A controlled trial of renal denervation for resistant hypertension. N. Engl. J. Med..

[55] Townsend R.R., Mahfoud F., Kandzari D.E., Kario K., Pocock S., Weber M.A., Ewen S., Tsioufis K., Tousoulis D., Sharp A.S.P. (2017). Catheter-based renal denervation in patients with uncontrolled hypertension in the absence of antihypertensive medications (SPYRAL HTN-OFF MED): A randomised, sham-controlled, proof-of-concept trial. Lancet.

[56] Azizi M., Schmieder R.E., Mahfoud F., Weber M.A., Daemen J., Davies J., Basile J., Kirtane A.J., Wang Y., Lobo M.D. (2018). Endovascular ultrasound renal denervation to treat hypertension (RADIANCE-HTN SOLO): A multicentre, international, single-blind, randomised, sham-controlled trial. Lancet.

[57] Kandzari D.E., Böhm M., Mahfoud F., Townsend R.R., Weber M.A., Pocock S., Tsioufis K., Tousoulis D., Choi J.W., East C. (2018). Effect of renal denervation on blood pressure in the presence of antihypertensive drugs: 6-month efficacy and safety results from the SPYRAL HTN-ON MED proof-of-concept randomised trial. Lancet.

[58] Hoppe U.C., Brandt M.C., Wachter R., Beige J., Rump L.C., Kroon A.A., Cates A.W., Lovett E.G., Haller H. (2012). Minimally invasive system for baroreflex activation therapy chronically lowers blood pressure with pacemaker-like safety profile: Results from the Barostim neo trial. J. Am. Soc. Hypertens..

[59] Scheffers I.J., Kroon A.A., Schmidli J., Jordan J., Tordoir J.J., Mohaupt M.G., Luft F.C., Haller H., Menne J., Engeli S. (2010). Novel baroreflex activation therapy in resistant hypertension: Results of a European multi-center feasibility study. J. Am. Coll. Cardiol..

[60] Bisognano J.D., Bakris G., Nadim M.K., Sanchez L., Kroon A.A., Schafer J., de Leeuw P.W., Sica D.A. (2011). Baroreflex activation therapy lowers blood pressure in patients with resistant hypertension: Results from the double-blind, randomized, placebo-controlled rheos pivotal trial. J. Am. Coll. Cardiol..

[61] Clinical Trials [Internet] (2015). Controlling and Lowering Blood Pressure with the MOBIUSHD™ (CALM-FIM_US).

[62] Wei F.F., Zhang Z.Y., Huang Q.F., Yang W.Y., Staessen J.A. (2018). Resistant hypertension. Kardiol. Pol..

[63] Scholz S.S., Vukadinović D., Lauder L., Ewen S., Ukena C., Townsend R.R., Wagenpfeil S., Böhm M., Mahfoud F. (2019). Effects of Arteriovenous Fistula on Blood Pressure in Patients With End-Stage Renal Disease: A Systematic Meta-Analysis. J. Am. Heart Assoc..

[64] Lobo M.D., Ott C., Sobotka P.A., Saxena M., Stanton A., Cockcroft J.R., Sulke N., Dolan E., van der Giet M., Hoyer J. (2017). Central Iliac Arteriovenous Anastomosis for Uncontrolled Hypertension: One-Year Results From the ROX CONTROL HTN Trial. Hypertension.

[65] Mathew R.O., Fleg J., Rangaswami J., Cai B., Asif A., Sidhu M.S., Bangalore S. (2019). Effect of Arteriovenous Fistula Creation on Systolic and Diastolic Blood Pressure in Patients With Pre-dialysis Advanced Chronic Kidney Disease. Am. J. Hypertens..

[66] Faul J., Schoors D., Brouwers S., Scott B., Jerrentrup A., Galvin J., Luitjens S., Dolan E. (2014). Creation of an iliac arteriovenous shunt lowers blood pressure in chronic obstructive pulmonary disease patients with hypertension. J. Vasc. Surg..

[67] Lobo M.D., Sobotka P.A., Stanton A., Cockcroft J.R., Sulke N., Dolan E., van der Giet M., Hoyer J., Furniss S.S., Foran J.P. (2015). Central arteriovenous anastomosis for the treatment of patients with uncontrolled hypertension (the ROX CONTROL HTN study): A randomised controlled trial. Lancet.

